# *N*-[(3-Phenylsulfanyl-1-phenylsulfonyl-1*H*-indol-2-yl)methyl]acetamide

**DOI:** 10.1107/S1600536809012525

**Published:** 2009-04-10

**Authors:** S. Thenmozhi, A. SubbiahPandi, V. Dhayalan, A. K. MohanaKrishnan

**Affiliations:** a Department of Physics, Presidency College (Autonomous), Chennai 600 005, India; b Department of Organic Chemistry, University of Madras, Guindy Campus, Chennai 600 025, India

## Abstract

In the title compound, C_23_H_20_N_2_O_3_S_2_, the phenylsulfonyl ring and phenylthio ring  make dihedral angles of 66.5 (7) and 81.2 (6)°, respectively, with the indole unit. In the crystal, mol­ecules are linked into centrosymmetric dimers *via* pairs of N—H⋯O hydrogen bonds with graph-set motif *R*_2_^2^(14). The crystal structure is further stabilized by weak inter­molecular C—H⋯O and very weak C—H⋯π inter­actions.

## Related literature

For the biological activity of indole derivatives, see: Singh *et al.* (2000 ▶); Andreani *et al.* (2001 ▶); Quetin-Leclercq (1994 ▶); Mukhopadhyay *et al.* (1981 ▶); Taylor *et al.* (1999 ▶); Williams *et al.* (1993 ▶); Sivaraman *et al.* (1996 ▶). For a related structure, see: Ravishankar *et al.* (2005 ▶).
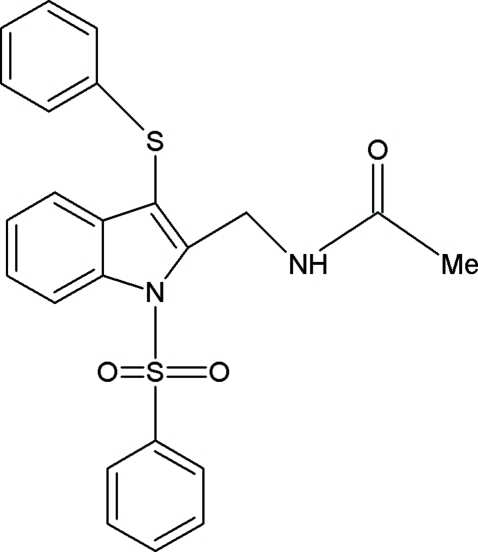


## Experimental

### 

#### Crystal data

C_23_H_20_N_2_O_3_S_2_
*M* *_r_* = 436.53
Triclinic, 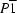

*a* = 8.8129 (2) Å
*b* = 10.8880 (3) Å
*c* = 11.3711 (3) Å
α = 86.698 (1)°
β = 76.494 (1)°
γ = 83.317 (1)°
*V* = 1053.21 (5) Å^3^
*Z* = 2
Mo *K*α radiation
μ = 0.28 mm^−1^
*T* = 293 K
0.21 × 0.19 × 0.17 mm

#### Data collection

Bruker Kappa APEXII CCD diffractometer
Absorption correction: multi-scan (*SADABS*; Sheldrick, 1996 ▶) *T*_min_ = 0.943, *T*_max_ = 0.953
28160 measured reflections
7167 independent reflections
5294 reflections with *I* > 2σ(*I*)
*R*_int_ = 0.026

#### Refinement

*R*[*F* ^2^ > 2σ(*F* ^2^)] = 0.041
*wR*(*F* ^2^) = 0.121
*S* = 0.98
7167 reflections
272 parameters
H-atom parameters constrained
Δρ_max_ = 0.33 e Å^−3^
Δρ_min_ = −0.39 e Å^−3^

### 

Data collection: *APEX2* (Bruker, 2004 ▶); cell refinement: *SAINT* (Bruker, 2004 ▶); data reduction: *SAINT*; program(s) used to solve structure: *SHELXS97* (Sheldrick, 2008 ▶); program(s) used to refine structure: *SHELXL97* (Sheldrick, 2008 ▶); molecular graphics: *ORTEP-3* (Farrugia, 1997 ▶); software used to prepare material for publication: *SHELXL97* and *PLATON* (Spek, 2009 ▶).

## Supplementary Material

Crystal structure: contains datablocks global, I. DOI: 10.1107/S1600536809012525/bt2922sup1.cif

Structure factors: contains datablocks I. DOI: 10.1107/S1600536809012525/bt2922Isup2.hkl

Additional supplementary materials:  crystallographic information; 3D view; checkCIF report

## Figures and Tables

**Table 1:** Hydrogen-bond geometry (Å, °)

*D*—H⋯*A*	*D*—H	H⋯*A*	*D*⋯*A*	*D*—H⋯*A*
N2—H2*A*⋯O2^i^	0.86	2.48	3.3179 (15)	165
C21—H21⋯O1^ii^	0.93	2.44	3.2666 (18)	149
C5—H5⋯*Cg*3^iii^	0.93	2.94	3.7634 (18)	149
C9—H9*A*⋯*Cg*4^iv^	0.97	2.95	3.5792 (16)	124
C11—H11*A*⋯*Cg*2^v^	0.96	2.91	3.5974 (21)	129
C16—H16⋯*Cg*4^vi^	0.93	2.95	3.7453 (21)	145

Symmetry codes: (i) 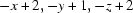; (ii) 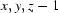; (iii) 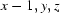; (iv) 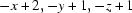; (v) 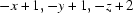; (vi) 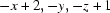. *Cg*2, *Cg*3 and C*g*4 are the centroids of the C1–C6, C12–C17 and C18–C23 rings, respectively.

**Fig. 1.**
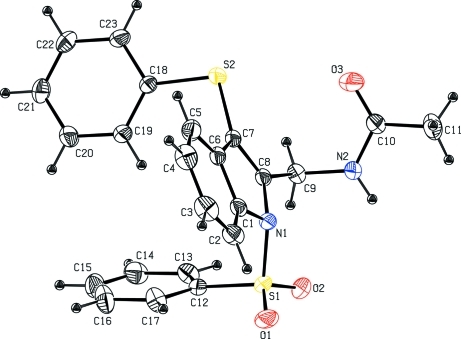
View of the title molecule with the atom labeling scheme. The displacement ellipsoids are drawn at the 30% probability level while the H atoms are shown as small spheres of arbitrary radii.

**Fig. 2.**
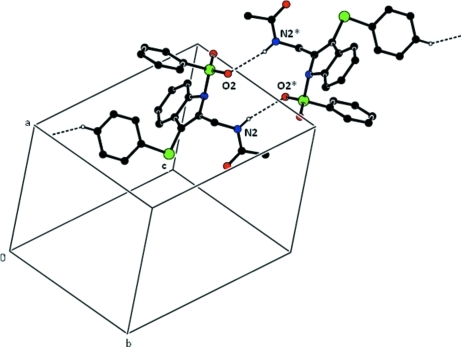
The crystal structure showing the centrosymmetric hydrogen bond motif R22(14). For the sake of clarity, the H atoms not involved in the motif have been omitted. The atoms marked with an asterisk (*) are at the symmetry position (-x, 1 - y, 2 - z). The dashed lines indicate the hydrogen bonds.

## supplementary crystallographic information

### Comment

Indole derivatives have been found to exhibit antibacterial, antifungal (Singh *et al.*, 2000) and antitumour activities (Andreani *et al.*, 2001). Some of the indole alkaloids extracted from plants possess interesting cytotoxic, antitumour or antiparasitic properties (Quetin-Leclercq, 1994; Mukhopadhyay *et al.*, 1981). Pyrido[1,2-*a*] indole derivatives have been identified as potent inhibitors of human immunodeficiency virus type 1 (Taylor *et al.*, 1999), and 5-chloro-3-(phenylsulfonyl) indole-2-carboxamide is reported to be a highly potent non-nucleoside inhibitor of HIV-1 reverse transcriptase (Williams *et al.*, 1993). The interaction of phenylsulfonylindole with calf thymus DNA has also been studied by spectroscopic methods (Sivaraman *et al.*, 1996). Against this background, and in order to obtain detailed information on molecular conformations in the solid state, X-ray studies of the title compound (Fig. 1) have been carried out.

The mean plane  of the indole ring system makes dihedral angles of 66.5 (7) and 81.2 (6)° with respect to the phenyl rings. The S–O, S–C, and S–N distances are 1.419 (11), 1.750 (13) and 1.679 (11) Å, respectively, these are comparable as observed in similar structures (Ravishankar *et al.*, 2005). As a result of the electron-withdrawing character of the phenylsulfonyl group, the N–C*sp*^2^ bond lengths, *viz*. N1–C1 [1.422 (9) Å] and N1–C8 [1.433 (6) Å], are longer than the mean value of 1.355 (14)Å  for N atoms with planar configurations.

Via N2–H2···O2 hydrogen bonds the molecules form cyclic centrosymmetric dimers [*R*_2_^2^(14)] shown in Fig.2. The structure is further stabilized by intermolecular C–H···π and C–H···O interactions as shown in Table. 1.

### Experimental

To a solution of 1-phenylsulfonyl-2-(bromomethyl)-3-(phenylthio)-1*H*-indole (0.5 g, 1.09 mmol) in dry acetonitrile (20 ml), ZnBr_2_ (0.49 g, 2.18 mmol), was added. The reaction mixture was then refluxed for 5 hr under N_2_ atmosphere. It was then poured over ice-water (30 ml) containing 1 ml of conc.HCl, extracted with CHCl_3_ (30 ml) and dried (Na_2_ SO_4_). Removal of solvent followed by crystallization from methanol afforded amide product. The amide was recrystallization from CDCl_3_. Single crystals of the title compound suitable for X-ray diffraction were obtained by slow evaporation of a solution in methanol.

### Refinement

All H atoms were fixed geometrically and allowed to ride on their parent C atoms, with C–H distances fixed in the range 0.93–0.97 Å with *U*_iso_(H) = 1.5*U*_eq_(C) for methyl H 1.2*U*_eq_(C) for other H atoms.

### Crystal data

**Table:**

C_23_H_20_N_2_O_3_S_2_	*Z* = 2
*M**_r_* = 436.53	*F*(000) = 456
Triclinic, *P*1	*D*_x_ = 1.377 Mg m^−^^3^
Hall symbol: -P 1	Mo *K*α radiation, λ = 0.71073 Å
*a* = 8.8129 (2) Å	Cell parameters from 7167 reflections
*b* = 10.8880 (3) Å	θ = 2.4–31.9°
*c* = 11.3711 (3) Å	µ = 0.28 mm^−^^1^
α = 86.698 (1)°	*T* = 293 K
β = 76.494 (1)°	Block, colourless
γ = 83.317 (1)°	0.21 × 0.19 × 0.17 mm
*V* = 1053.21 (5) Å^3^	

### Data collection

**Table:**

Bruker Kappa APEXII CCD diffractometer	7167 independent reflections
Radiation source: fine-focus sealed tube	5294 reflections with *I* > 2σ(*I*)
graphite	*R*_int_ = 0.026
ω scans	θ_max_ = 31.9°, θ_min_ = 2.4°
Absorption correction: multi-scan (*SADABS*; Sheldrick, 1996)	*h* = −13→13
*T*_min_ = 0.943, *T*_max_ = 0.953	*k* = −16→16
28160 measured reflections	*l* = −16→16

### Refinement

**Table:**

Refinement on *F*^2^	Primary atom site location: structure-invariant direct methods
Least-squares matrix: full	Secondary atom site location: difference Fourier map
*R*[*F*^2^ > 2σ(*F*^2^)] = 0.041	Hydrogen site location: inferred from neighbouring sites
*wR*(*F*^2^) = 0.121	H-atom parameters constrained
*S* = 0.98	*w* = 1/[σ^2^(*F*_o_^2^) + (0.0595*P*)^2^ + 0.2774*P*] where *P* = (*F*_o_^2^ + 2*F*_c_^2^)/3
7167 reflections	(Δ/σ)_max_ = 0.005
272 parameters	Δρ_max_ = 0.33 e Å^−^^3^
0 restraints	Δρ_min_ = −0.39 e Å^−^^3^

### Special details

**Table:**

Geometry. All e.s.d.'s (except the e.s.d. in the dihedral angle between two l.s. planes) are estimated using the full covariance matrix. The cell e.s.d.'s are taken into account individually in the estimation of e.s.d.'s in distances, angles and torsion angles; correlations between e.s.d.'s in cell parameters are only used when they are defined by crystal symmetry. An approximate (isotropic) treatment of cell e.s.d.'s is used for estimating e.s.d.'s involving l.s. planes.
Refinement. Refinement of *F*^2^ against ALL reflections. The weighted *R*-factor *wR* and goodness of fit *S* are based on *F*^2^, conventional *R*-factors *R* are based on *F*, with *F* set to zero for negative *F*^2^. The threshold expression of *F*^2^ > σ(*F*^2^) is used only for calculating *R*-factors(gt) *etc*. and is not relevant to the choice of reflections for refinement. *R*-factors based on *F*^2^ are statistically about twice as large as those based on *F*, and *R*- factors based on ALL data will be even larger.

### Fractional atomic coordinates and isotropic or equivalent isotropic displacement parameters (Å^2^)

**Table:**

	*x*	*y*	*z*	*U*_iso_*/*U*_eq_	
C1	0.80217 (15)	0.18662 (12)	0.83970 (11)	0.0347 (3)	
C2	0.79544 (18)	0.07935 (15)	0.91125 (14)	0.0470 (3)	
H2	0.8532	0.0644	0.9703	0.056*	
C3	0.6993 (2)	−0.00414 (16)	0.89092 (17)	0.0567 (4)	
H3	0.6929	−0.0775	0.9370	0.068*	
C4	0.6116 (2)	0.01768 (16)	0.80390 (17)	0.0561 (4)	
H4	0.5467	−0.0404	0.7937	0.067*	
C5	0.61930 (18)	0.12411 (15)	0.73246 (14)	0.0465 (3)	
H5	0.5611	0.1385	0.6736	0.056*	
C6	0.71616 (15)	0.20927 (12)	0.75069 (11)	0.0348 (3)	
C7	0.74318 (15)	0.33121 (12)	0.69884 (11)	0.0338 (3)	
C8	0.83792 (15)	0.38163 (12)	0.75660 (11)	0.0329 (2)	
C9	0.87341 (16)	0.51288 (13)	0.75136 (13)	0.0393 (3)	
H9A	0.9854	0.5152	0.7415	0.047*	
H9B	0.8427	0.5560	0.6822	0.047*	
C10	0.64041 (17)	0.62259 (15)	0.87510 (15)	0.0463 (3)	
C11	0.5609 (2)	0.66919 (19)	0.99829 (18)	0.0619 (5)	
H11A	0.4948	0.7443	0.9901	0.093*	
H11B	0.6387	0.6850	1.0405	0.093*	
H11C	0.4982	0.6081	1.0430	0.093*	
C12	1.17375 (14)	0.22315 (13)	0.72453 (12)	0.0355 (3)	
C13	1.23409 (17)	0.30613 (15)	0.63391 (14)	0.0458 (3)	
H13	1.2222	0.3904	0.6477	0.055*	
C14	1.31247 (19)	0.26126 (19)	0.52229 (16)	0.0560 (4)	
H14	1.3537	0.3157	0.4601	0.067*	
C15	1.3295 (2)	0.1370 (2)	0.50313 (16)	0.0602 (5)	
H15	1.3821	0.1075	0.4277	0.072*	
C16	1.2700 (2)	0.05551 (18)	0.59387 (19)	0.0656 (5)	
H16	1.2830	−0.0288	0.5798	0.079*	
C17	1.19143 (19)	0.09758 (15)	0.70519 (16)	0.0517 (4)	
H17	1.1506	0.0425	0.7669	0.062*	
C18	0.76928 (15)	0.32193 (12)	0.45609 (11)	0.0336 (3)	
C19	0.90885 (16)	0.24822 (14)	0.45385 (12)	0.0417 (3)	
H19	0.9484	0.2367	0.5232	0.050*	
C20	0.98919 (19)	0.19193 (16)	0.34812 (14)	0.0503 (4)	
H20	1.0825	0.1418	0.3468	0.060*	
C21	0.9327 (2)	0.20926 (16)	0.24488 (14)	0.0512 (4)	
H21	0.9882	0.1719	0.1738	0.061*	
C22	0.7938 (2)	0.28199 (17)	0.24709 (13)	0.0524 (4)	
H22	0.7551	0.2934	0.1774	0.063*	
C23	0.71127 (17)	0.33808 (15)	0.35210 (13)	0.0440 (3)	
H23	0.6169	0.3867	0.3533	0.053*	
N1	0.87779 (12)	0.29375 (10)	0.84621 (9)	0.0346 (2)	
N2	0.78974 (13)	0.57402 (11)	0.86117 (11)	0.0403 (3)	
H2A	0.8371	0.5792	0.9185	0.048*	
O1	1.06998 (14)	0.18125 (12)	0.95384 (10)	0.0559 (3)	
O2	1.09978 (14)	0.39630 (11)	0.88318 (11)	0.0552 (3)	
O3	0.57273 (17)	0.62503 (18)	0.79379 (14)	0.0910 (5)	
S1	1.06294 (4)	0.27656 (3)	0.86411 (3)	0.03906 (10)	
S2	0.65950 (4)	0.40353 (3)	0.58434 (3)	0.04266 (10)	

### Atomic displacement parameters (Å^2^)

**Table:**

	*U*^11^	*U*^22^	*U*^33^	*U*^12^	*U*^13^	*U*^23^
C1	0.0339 (6)	0.0363 (7)	0.0294 (6)	0.0033 (5)	−0.0017 (5)	−0.0011 (5)
C2	0.0472 (8)	0.0473 (8)	0.0409 (8)	0.0023 (6)	−0.0049 (6)	0.0087 (6)
C3	0.0589 (10)	0.0442 (9)	0.0590 (10)	−0.0056 (7)	−0.0003 (8)	0.0117 (7)
C4	0.0557 (9)	0.0456 (9)	0.0646 (11)	−0.0137 (7)	−0.0041 (8)	−0.0036 (8)
C5	0.0465 (8)	0.0474 (8)	0.0459 (8)	−0.0054 (6)	−0.0093 (6)	−0.0084 (6)
C6	0.0363 (6)	0.0365 (7)	0.0288 (6)	0.0022 (5)	−0.0039 (5)	−0.0048 (5)
C7	0.0373 (6)	0.0350 (6)	0.0270 (5)	0.0056 (5)	−0.0072 (5)	−0.0034 (5)
C8	0.0341 (6)	0.0346 (6)	0.0268 (5)	0.0043 (5)	−0.0043 (4)	−0.0025 (5)
C9	0.0388 (6)	0.0359 (7)	0.0403 (7)	−0.0002 (5)	−0.0049 (5)	−0.0030 (5)
C10	0.0393 (7)	0.0476 (8)	0.0508 (8)	0.0029 (6)	−0.0097 (6)	−0.0097 (7)
C11	0.0522 (9)	0.0638 (11)	0.0635 (11)	0.0005 (8)	0.0013 (8)	−0.0246 (9)
C12	0.0307 (6)	0.0404 (7)	0.0362 (6)	−0.0001 (5)	−0.0105 (5)	−0.0041 (5)
C13	0.0396 (7)	0.0441 (8)	0.0503 (8)	−0.0011 (6)	−0.0060 (6)	0.0022 (6)
C14	0.0432 (8)	0.0730 (12)	0.0446 (8)	−0.0027 (7)	0.0002 (6)	0.0076 (8)
C15	0.0448 (8)	0.0834 (14)	0.0490 (9)	−0.0026 (8)	−0.0006 (7)	−0.0234 (9)
C16	0.0572 (10)	0.0559 (11)	0.0783 (13)	−0.0077 (8)	0.0028 (9)	−0.0295 (10)
C17	0.0490 (8)	0.0422 (8)	0.0576 (9)	−0.0052 (6)	0.0015 (7)	−0.0055 (7)
C18	0.0365 (6)	0.0355 (6)	0.0295 (6)	−0.0043 (5)	−0.0093 (5)	0.0010 (5)
C19	0.0411 (7)	0.0508 (8)	0.0330 (6)	0.0029 (6)	−0.0113 (5)	−0.0029 (6)
C20	0.0460 (8)	0.0584 (10)	0.0411 (8)	0.0068 (7)	−0.0043 (6)	−0.0064 (7)
C21	0.0606 (9)	0.0569 (10)	0.0322 (7)	−0.0065 (7)	−0.0015 (6)	−0.0070 (6)
C22	0.0645 (10)	0.0649 (10)	0.0310 (7)	−0.0076 (8)	−0.0170 (7)	−0.0010 (7)
C23	0.0449 (7)	0.0534 (9)	0.0355 (7)	−0.0003 (6)	−0.0163 (6)	0.0022 (6)
N1	0.0343 (5)	0.0400 (6)	0.0282 (5)	0.0025 (4)	−0.0076 (4)	−0.0017 (4)
N2	0.0391 (6)	0.0397 (6)	0.0439 (6)	0.0019 (5)	−0.0137 (5)	−0.0120 (5)
O1	0.0594 (7)	0.0743 (8)	0.0344 (5)	0.0054 (6)	−0.0204 (5)	0.0089 (5)
O2	0.0576 (6)	0.0579 (7)	0.0580 (7)	−0.0021 (5)	−0.0261 (5)	−0.0220 (5)
O3	0.0563 (8)	0.1470 (15)	0.0704 (9)	0.0362 (9)	−0.0318 (7)	−0.0335 (9)
S1	0.04034 (17)	0.0485 (2)	0.03049 (16)	0.00304 (14)	−0.01514 (13)	−0.00580 (13)
S2	0.04688 (19)	0.0454 (2)	0.03417 (17)	0.01400 (14)	−0.01465 (14)	−0.00459 (14)

### Geometric parameters (Å, °)

**Table:**

C1—C2	1.3834 (19)	C12—S1	1.7496 (13)
C1—C6	1.3949 (18)	C13—C14	1.382 (2)
C1—N1	1.4226 (18)	C13—H13	0.9300
C2—C3	1.376 (2)	C14—C15	1.368 (3)
C2—H2	0.9300	C14—H14	0.9300
C3—C4	1.385 (3)	C15—C16	1.370 (3)
C3—H3	0.9300	C15—H15	0.9300
C4—C5	1.375 (2)	C16—C17	1.369 (2)
C4—H4	0.9300	C16—H16	0.9300
C5—C6	1.386 (2)	C17—H17	0.9300
C5—H5	0.9300	C18—C19	1.3840 (18)
C6—C7	1.4439 (19)	C18—C23	1.3885 (17)
C7—C8	1.3543 (18)	C18—S2	1.7727 (13)
C7—S2	1.7477 (12)	C19—C20	1.382 (2)
C8—N1	1.4332 (16)	C19—H19	0.9300
C8—C9	1.4930 (19)	C20—C21	1.374 (2)
C9—N2	1.4482 (17)	C20—H20	0.9300
C9—H9A	0.9700	C21—C22	1.375 (2)
C9—H9B	0.9700	C21—H21	0.9300
C10—O3	1.2093 (19)	C22—C23	1.379 (2)
C10—N2	1.3368 (18)	C22—H22	0.9300
C10—C11	1.500 (2)	C23—H23	0.9300
C11—H11A	0.9600	N1—S1	1.6789 (11)
C11—H11B	0.9600	N2—H2A	0.8600
C11—H11C	0.9600	O1—S1	1.4193 (11)
C12—C13	1.382 (2)	O2—S1	1.4198 (12)
C12—C17	1.382 (2)		
			
C2—C1—C6	121.75 (14)	C14—C13—H13	120.7
C2—C1—N1	130.00 (13)	C15—C14—C13	120.14 (16)
C6—C1—N1	108.02 (11)	C15—C14—H14	119.9
C3—C2—C1	116.88 (15)	C13—C14—H14	119.9
C3—C2—H2	121.6	C14—C15—C16	120.71 (16)
C1—C2—H2	121.6	C14—C15—H15	119.6
C2—C3—C4	122.07 (15)	C16—C15—H15	119.6
C2—C3—H3	119.0	C17—C16—C15	120.32 (17)
C4—C3—H3	119.0	C17—C16—H16	119.8
C5—C4—C3	120.89 (16)	C15—C16—H16	119.8
C5—C4—H4	119.6	C16—C17—C12	119.01 (16)
C3—C4—H4	119.6	C16—C17—H17	120.5
C4—C5—C6	118.11 (15)	C12—C17—H17	120.5
C4—C5—H5	120.9	C19—C18—C23	119.60 (13)
C6—C5—H5	120.9	C19—C18—S2	123.85 (10)
C5—C6—C1	120.29 (13)	C23—C18—S2	116.52 (10)
C5—C6—C7	132.35 (13)	C20—C19—C18	119.66 (13)
C1—C6—C7	107.20 (12)	C20—C19—H19	120.2
C8—C7—C6	109.04 (11)	C18—C19—H19	120.2
C8—C7—S2	125.89 (11)	C21—C20—C19	120.68 (15)
C6—C7—S2	125.02 (10)	C21—C20—H20	119.7
C7—C8—N1	108.48 (11)	C19—C20—H20	119.7
C7—C8—C9	128.32 (12)	C20—C21—C22	119.72 (14)
N1—C8—C9	122.13 (11)	C20—C21—H21	120.1
N2—C9—C8	110.29 (11)	C22—C21—H21	120.1
N2—C9—H9A	109.6	C21—C22—C23	120.40 (14)
C8—C9—H9A	109.6	C21—C22—H22	119.8
N2—C9—H9B	109.6	C23—C22—H22	119.8
C8—C9—H9B	109.6	C22—C23—C18	119.92 (13)
H9A—C9—H9B	108.1	C22—C23—H23	120.0
O3—C10—N2	121.55 (15)	C18—C23—H23	120.0
O3—C10—C11	122.30 (15)	C1—N1—C8	107.23 (10)
N2—C10—C11	116.11 (14)	C1—N1—S1	119.17 (9)
C10—C11—H11A	109.5	C8—N1—S1	118.64 (9)
C10—C11—H11B	109.5	C10—N2—C9	121.31 (12)
H11A—C11—H11B	109.5	C10—N2—H2A	119.3
C10—C11—H11C	109.5	C9—N2—H2A	119.3
H11A—C11—H11C	109.5	O1—S1—O2	119.66 (7)
H11B—C11—H11C	109.5	O1—S1—N1	106.41 (7)
C13—C12—C17	121.18 (14)	O2—S1—N1	106.61 (6)
C13—C12—S1	120.19 (11)	O1—S1—C12	108.97 (7)
C17—C12—S1	118.52 (12)	O2—S1—C12	110.17 (7)
C12—C13—C14	118.64 (15)	N1—S1—C12	103.77 (6)
C12—C13—H13	120.7	C7—S2—C18	101.32 (6)
			
C6—C1—C2—C3	−0.2 (2)	C19—C20—C21—C22	−0.9 (3)
N1—C1—C2—C3	173.51 (14)	C20—C21—C22—C23	0.3 (3)
C1—C2—C3—C4	−0.6 (2)	C21—C22—C23—C18	0.5 (2)
C2—C3—C4—C5	1.0 (3)	C19—C18—C23—C22	−0.8 (2)
C3—C4—C5—C6	−0.5 (2)	S2—C18—C23—C22	177.16 (12)
C4—C5—C6—C1	−0.3 (2)	C2—C1—N1—C8	−175.59 (13)
C4—C5—C6—C7	−175.17 (14)	C6—C1—N1—C8	−1.19 (13)
C2—C1—C6—C5	0.7 (2)	C2—C1—N1—S1	46.01 (18)
N1—C1—C6—C5	−174.30 (12)	C6—C1—N1—S1	−139.59 (9)
C2—C1—C6—C7	176.72 (12)	C7—C8—N1—C1	0.11 (13)
N1—C1—C6—C7	1.76 (14)	C9—C8—N1—C1	169.22 (11)
C5—C6—C7—C8	173.66 (14)	C7—C8—N1—S1	138.76 (10)
C1—C6—C7—C8	−1.72 (14)	C9—C8—N1—S1	−52.12 (14)
C5—C6—C7—S2	−3.8 (2)	O3—C10—N2—C9	−5.2 (3)
C1—C6—C7—S2	−179.22 (9)	C11—C10—N2—C9	172.65 (14)
C6—C7—C8—N1	0.98 (14)	C8—C9—N2—C10	−83.42 (16)
S2—C7—C8—N1	178.45 (9)	C1—N1—S1—O1	−43.85 (11)
C6—C7—C8—C9	−167.25 (12)	C8—N1—S1—O1	−177.59 (10)
S2—C7—C8—C9	10.2 (2)	C1—N1—S1—O2	−172.62 (10)
C7—C8—C9—N2	104.47 (15)	C8—N1—S1—O2	53.64 (11)
N1—C8—C9—N2	−62.33 (15)	C1—N1—S1—C12	71.05 (10)
C17—C12—C13—C14	0.4 (2)	C8—N1—S1—C12	−62.69 (11)
S1—C12—C13—C14	−175.91 (12)	C13—C12—S1—O1	−156.18 (11)
C12—C13—C14—C15	−0.2 (2)	C17—C12—S1—O1	27.46 (13)
C13—C14—C15—C16	−0.2 (3)	C13—C12—S1—O2	−23.05 (13)
C14—C15—C16—C17	0.4 (3)	C17—C12—S1—O2	160.59 (12)
C15—C16—C17—C12	−0.2 (3)	C13—C12—S1—N1	90.75 (12)
C13—C12—C17—C16	−0.2 (2)	C17—C12—S1—N1	−85.61 (12)
S1—C12—C17—C16	176.18 (14)	C8—C7—S2—C18	108.39 (12)
C23—C18—C19—C20	0.2 (2)	C6—C7—S2—C18	−74.53 (12)
S2—C18—C19—C20	−177.54 (12)	C19—C18—S2—C7	−15.37 (14)
C18—C19—C20—C21	0.6 (2)	C23—C18—S2—C7	166.79 (11)

### Hydrogen-bond geometry (Å, °)

**Table:**

*D*—H···*A*	*D*—H	H···*A*	*D*···*A*	*D*—H···*A*
N2—H2A···O2^i^	0.86	2.48	3.3179 (15)	165
C21—H21···O1^ii^	0.93	2.44	3.2666 (18)	149
C5—H5···Cg3^iii^	0.93	2.94	3.7634 (18)	149
C9—H9A···Cg4^iv^	0.97	2.95	3.5792 (16)	124
C11—H11A···Cg2^v^	0.96	2.91	3.5974 (21)	129
C16—H16···Cg4^vi^	0.93	2.95	3.7453 (21)	145

Symmetry codes: (i) −*x*+2, −*y*+1, −*z*+2; (ii) *x*, *y*, *z*−1; (iii) *x*−1, *y*, *z*; (iv) −*x*+2, −*y*+1, −*z*+1; (v) −*x*+1, −*y*+1, −*z*+2; (vi) −*x*+2, −*y*, −*z*+1.

## References

[bb1] Andreani, A., Granaiola, M., Leoni, A., Locatelli, A., Morigi, R., Rambaldi, M., Giorgi, G., Salvini, L. & Garaliene, V. (2001). *Anticancer Drug Des.* **16**, 167–174. 11962514

[bb2] Bruker (2004). *APEX2* and *SAINT* Bruker AXS Inc., Madison, Wisconsin, USA.

[bb3] Farrugia, L. J. (1997). *J. Appl. Cryst.* **30**, 565.

[bb4] Mukhopadhyay, S., Handy, G. A., Funayama, S. & Cordell, G. A. (1981). *J. Nat. Prod.* **44**, 696–700. 10.1021/np50018a014 7334384

[bb5] Quetin-Leclercq, J. (1994). *J. Pharm. Belg.* **49**, 181–192. 8057233

[bb6] Ravishankar, T., Chinnakali, K., Arumugam, N., Srinivasan, P. C., Usman, A. & Fun, H.-K. (2005). *Acta Cryst.* E**61**, o2455–o2457. 10.1107/s0108270103001410 12711788

[bb7] Sheldrick, G. M. (1996). *SADABS* University of Gottingen, Germany.

[bb8] Sheldrick, G. M. (2008). *Acta Cryst.* A**64**, 112–122. 10.1107/S0108767307043930 18156677

[bb9] Singh, U. P., Sarma, B. K., Mishra, P. K. & Ray, A. B. (2000). *Folia Microbiol.* **45**, 173–176. 10.1007/BF02817419 11271828

[bb10] Sivaraman, J., Subramanian, K., Velmurugan, D., Subramanian, E. & Seetharaman, J. (1996). *J. Mol. Struct.* **385**, 123–128.

[bb11] Spek, A. L. (2009). *Acta Cryst.* D**65**, 148–155. 10.1107/S090744490804362X PMC2631630 19171970

[bb12] Taylor, D. L., Ahmed, P. S., Chambers, P., Tyms, A. S., Bedard, J., Duchaine, J., Falardeau, G., Lavallee, J. F., Brown, W., Rando, R. F. & Bowlin, T. (1999). *Antiviral Chem. Chemother.* **10**, 79–86. 10.1177/095632029901000204 10335402

[bb13] Williams, T. M., Ciccarone, T. M., MacTough, S. C., Rooney, C. S., Balani, S. K., Condra, J. H., Emini, E. A., Goldman, M. E., Greenlee, W. J. & Kauffman, L. R. (1993). *J. Med. Chem.* **36**, 1291–1294. 10.1021/jm00061a022 7683725

